# Parapipe: a pipeline for parasite next-generation sequencing data analysis applied to Cryptosporidium

**DOI:** 10.1099/acmi.0.000993.v3

**Published:** 2025-08-08

**Authors:** Arthur V. Morris, Guy Robinson, Rachel Chalmers, Simone Cacciò, Thomas Connor

**Affiliations:** 1 Cardiff School of Biosciences, Cardiff University, Cardiff, UK; 2 Cryptosporidium Reference Unit, Public Health Wales, Singleton Hospital, Swansea, UK; 3 Department of Infectious Diseases, Istituto Superiore Di Sanita, Rome, Italy; 4 Pathogen Genomics Unit (PenGU), Public Health Wales, Wales Genomic Health Centre, Cardiff, UK

**Keywords:** bioinformatics, *Cryptosporidium*, epidemiology, genomics, Parapipe, parasite

## Abstract

*Cryptosporidium*, a protozoan parasite of significant public health concern, is responsible for severe diarrhoeal disease, particularly in immunocompromised individuals and young children in resource-limited settings. Analysis of whole-genome next-generation sequencing (NGS) data is critical in improving our understanding of *Cryptosporidium* epidemiology, transmission and diversity. However, effective analysis of NGS data in a public health context necessitates the development of robust, validated computational tools. We present Parapipe, an ISO-accreditable bioinformatic pipeline for high-throughput analysis of NGS data from *Cryptosporidium* and related taxa. Built using Nextflow DSL2 and containerized with Singularity, Parapipe is modular, portable, scalable and designed for use by public health laboratories. Using both simulated and real *Cryptosporidium* datasets, we demonstrate the power of Parapipe’s genomic analysis for generating epidemiological insights. We highlight how whole-genome analysis yields substantially greater phylogenetic resolution than conventional *gp60* molecular typing in *Cryptosporidium parvum*. Uniquely, Parapipe facilitates the integration of mixed infection analysis and phylogenomic clustering with epidemiological metadata, representing a powerful tool in the investigation of complex transmission pathways and identification of outbreak sources. Parapipe significantly advances genomic surveillance of *Cryptosporidium*, offering a streamlined, reproducible analytical framework. By automating a complex workflow and delivering detailed genomic characterization, Parapipe provides a valuable tool for public health agencies and researchers, supporting efforts to mitigate the global burden of cryptosporidiosis.

## Graphical abstract

An outline of Parapipe's workflow, illustrating its five core modules: quality control, variant calling, polyclonality detection, clustering, and report generation.

**Impact Statement**

*Cryptosporidium* is a eukaryotic pathogen responsible for over 200,000 deaths annually. Due to a lack of effective drugs or vaccines, control is dependent on understanding the genetic diversity of this parasite to reveal transmission patterns and interrupt spread. Current molecular typing methods are limited in their ability to resolve genetic diversity, hindering effective surveillance and outbreak response. This study introduces Parapipe, the first publicly available bioinformatic pipeline for analysing eukaryotic pathogen next-generation sequencing data. Parapipe arms public health and research laboratories with unprecedented precision to carry out epidemiological surveillance of eukaryotic pathogens, addressing critical gaps in the field. Uniquely, Parapipe automates the characterization of m.o.i., a phenomenon where hosts carry multiple genetic populations of a pathogen, which often confounds molecular surveillance methods. By integrating m.o.i. analysis with phylogenomic clustering, Parapipe offers deeper insights into transmission pathways of eukaryotic pathogens.Developed in collaboration with leading European public health laboratories, Parapipe represents a transformative tool with utility in both eukaryotic pathogen research and public health surveillance and establishes the computational foundation for replacing molecular typing schemes with high-resolution genomic surveillance. Its modularity and ISO accreditability ensure rapid adoption into surveillance programmes, marking an important milestone in the fight against *Cryptosporidium* and related pathogens worldwide.

## Data Summary

The source code for Parapipe is available at https://github.com/ArthurVM/Parapipe. The clonal and heterogeneous lineage definition files, along with scripts used to generate lineage NGS datasets, are available at https://github.com/ArthurVM/Parapipe_test_data. The source code for pyMOI is available at https://github.com/ArthurVM/PyMOI. Scripts for simulating NGS reads using ART can be found at https://github.com/ArthurVM/ngsContrive.

The authors confirm that all supporting data, code and protocols have been provided within the article or through supplementary data files.

## Introduction

*Cryptosporidium* is a genus of protozoan parasites within the phylum Apicomplexa and is recognized as a major cause of diarrhoeal disease in mammals worldwide. In low-resource settings across Asia and sub-Saharan Africa, the parasite is estimated to cause over 200,000 deaths annually among young children [1]. Young, malnourished or otherwise immunocompromised individuals are at particular risk of significant morbidity and mortality [2]. Humans acquire the infection faecal-orally through multiple routes and vehicles, including direct human-to-human and animal-to-human contacts and consumption of contaminated water and food.

Although at least 48 species of *Cryptosporidium* have been described, infection in humans is mostly caused by two species: the zoonotic *Cryptosporidium parvum* and the anthroponotic *Cryptosporidium hominis*. Currently, there are no truly effective drugs or vaccines to treat or prevent cryptosporidiosis, and thus control is heavily dependent on the prevention of infection, which in turn requires a detailed understanding of *Cryptosporidium* epidemiology, population structure and transmission dynamics [1]. In this context, genomic analysis could provide a cornerstone for the control and prevention of cryptosporidiosis, as it allows for high-resolution typing evaluation, beyond that which is possible using conventional molecular techniques. However, a pre-requisite for the routine use of genomics for human and animal public health purposes necessitates the development of reliable bioinformatic pipelines, validated to a high standard, which enables data analysis to be completed in a short space of time. The timely development of an appropriate pipeline coincides with technologies enabling next-generation sequencing (NGS) of *Cryptosporidium* from DNA extracted directly from stools using hybridization baits [2,3]. Previously, only limited numbers of specifically selected specimens have been genome sequenced from the oocyst (transmissive) stage of this practically non-culturable organism that is present in low density in complex matrices such as faeces [4]. *C. parvum* is conventionally subtyped by interrogating fragment length and sequence variation across a highly polymorphic variable number tandem repeat (VNTR) region within a surface glycoprotein gene, *gp60* [5]. This locus has been utilized for *Cryptosporidium* subtyping for over two decades and provides the only standardized subtyping scheme for *Cryptosporidium* [6]. However, despite limitations of *gp60* subtyping (single locus, usually by Sanger sequencing), its undeniable utility, comparatively low cost, wet lab simplicity and the familiarity of this subtyping scheme within the wider *Cryptosporidium* community have encouraged its continuing use [4,7]. Yet there is evidence that higher discrimination, improved epidemiological concordance, identification of m.o.i. and greater public health utility can be provided by multilocus genotyping [6,8].

Here, we present Parapipe, a bioinformatic pipeline that has been developed to a standard that would allow it to be accredited as part of an ISO 15189 or 17025 laboratory process and meet the standards and requirements of public health microbiological investigations [1]. Parapipe has been developed for *Cryptosporidium* to meet the needs of clinical laboratories and is intended to solve the issues of NGS data quality assessment and control, mapping, variant calling, m.o.i. investigation and clustering analysis. Following the bioinformatics approach adopted for other accredited genomics services within Public Health Wales, we have developed a modular system in which Parapipe is subdivided into functional components that can be tested individually as well as part of the larger whole. We carry out extensive module and end-to-end testing to demonstrate pipeline reliability and use Parapipe to investigate the heterogeneity and relatedness of a *C. parvum* NGS dataset.

Parapipe represents the first publicly available pipeline designed to carry out essential pre-processing and analysis of *Cryptosporidium* NGS data. Although Parapipe was designed with *Cryptosporidium* in mind, and its modular architecture permits adaptation, its current implementation and validation procedure have been tailored to organisms with similar genomic characteristics (haploid, compact genomes with low repeat content and low heterozygosity). It is currently only validated for use on *Cryptosporidium* and designed for use with Illumina short-read data.

## Methods

### Implementation

Parapipe is a command line-based pipeline written in Nextflow DSL2 [9], allowing for modularization and containerization using Singularity [10]. The use of Nextflow and Singularity ensures that the software is portable between systems and enables a simplified process for installing and running the pipeline, either using on-premises systems or on a commercial cloud. Parapipe is built using well-established read pre-processing, quality control, mapping and variant calling tools. Parapipe is a linear pipeline whereby each module is executed in a prescribed order on every sample.

As input data, it takes a set of paired-end reads in FASTQ format. Each read set is passed through the pipeline as an individual run, allowing multiple read sets to be processed in a parallel manner. Reference datasets (consisting of a sequence and annotation pair) can be deposited in a subdirectory within the Parapipe root directory for convenient use by the user via a command line argument.

**Fig. 1.**
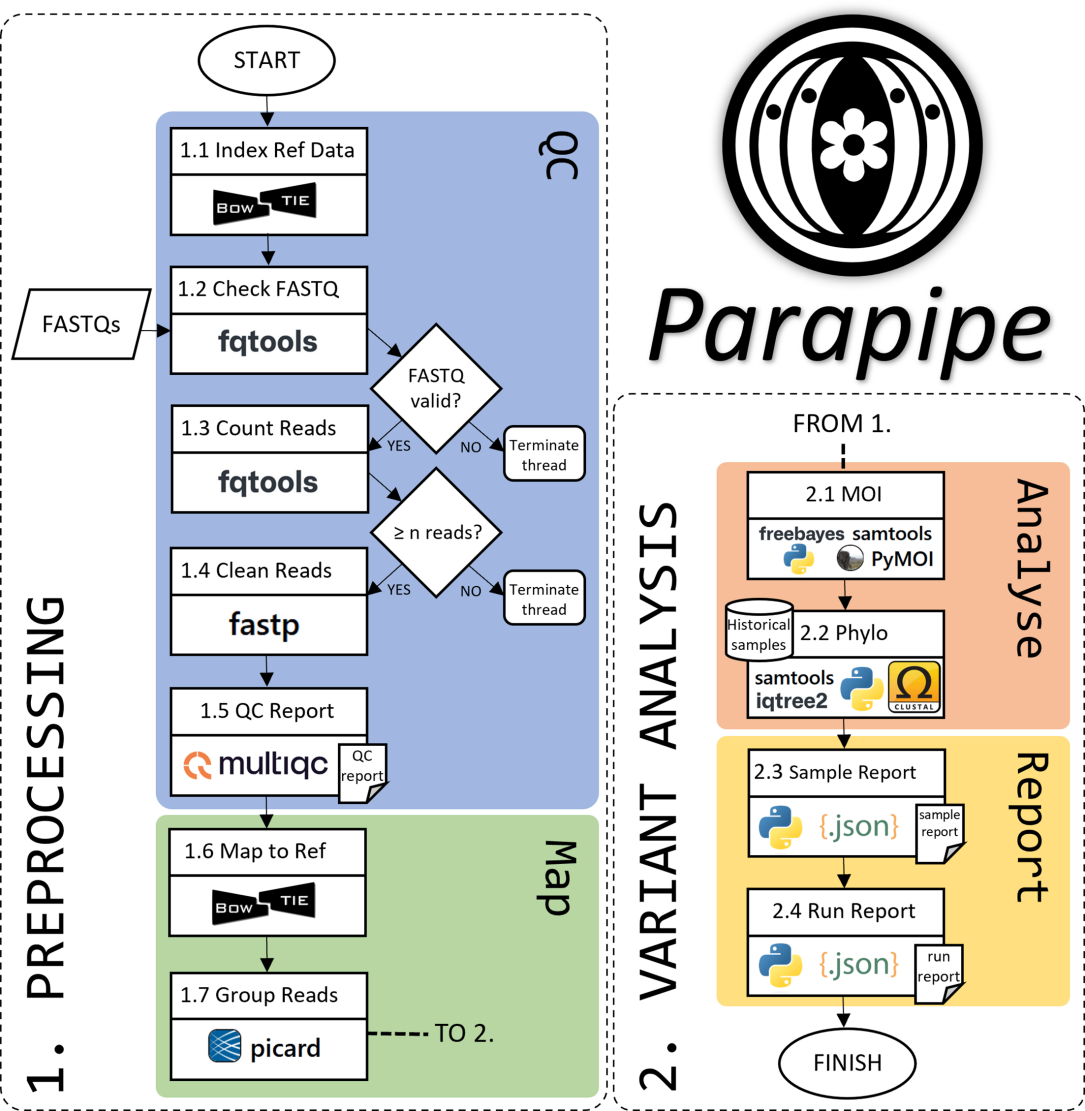
Workflow schematic for Parapipe, detailing the processes that comprise the two primary work modules within the pipeline, pre-processing and variant analysis.

Parapipe consists of two primary work modules, comprising twelve individual processes (1.1–1.8 and 2.1–2.4; see Fig. 1). The workflow from module 1 to module 2 is automated. The first module prepares the reference data and performs quality control, pre-processing and mapping on the input reads. The second module performs variant calling, clustering, phylogenetic and phylogenomic analysis.

#### Module 1

Module 1 consists of eight processes with two logic gates. Process 1.1 fetches and prepares the reference files by constructing a Bowtie2 index and a samtools faidx index from the reference FASTA file [11,12]. FASTQ files are then checked using fqtools (processes 1.2 and 1.3) to ensure the input FASTQ files are valid and contain a sufficient number of paired reads [13]. Logic gates exist to evaluate the output of fqtools. FASTQ file sets that do not pass these checks are not processed further, and the relevant threads are terminated. Processes 1.4–1.5 perform cleaning, trimming and quality control of the input FASTQ sets using fastp and fastQC and aggregate all QC reports from all FASTQ sets in this Parapipe run using multiQC [14,15]. Read mapping, deduplication and group assignment are carried out using Bowtie2 and Picard, respectively, during processes 1.6 and 1.7 [16]. Since the input read sets constitute a single run of an isolate, all reads in each sample FASTQ set are assigned to the same read group. This is a necessary step to facilitate downstream variant and sample heterogeneity analysis.

The default read number threshold required for analysis is 1 million paired reads. This parameter can be adjusted by the user. The minimum quality threshold for short-read data is hard-coded to ensure sensitive and reliable results from SNP detection, m.o.i. analysis and phylogenetic analysis. Reads are filtered with the following parameters: a minimum read length of 50 bases (--length_required 50), a minimum average quality score of 10 (--average_qual 10) and removal of low-complexity reads (--low_complexity_filter). Base correction in overlapping regions was enabled (--correction), and aggressive quality trimming was applied at both ends of reads (--cut_right and --cut_tail). Tail trimming uses a sliding window of size 1 (--cut_tail_window_size 1), with bases removed if their average quality within the window falls below 20 (--cut_tail_mean_quality 20).

#### Module 2

Module 2 carries out the variant analysis and comparative genomics within Parapipe. Process 2.1 carries out m.o.i. investigation to elucidate the extent to which multiple distinct genomic variants exist within the dataset. The m.o.i. can occur as a result of either a mixed infection or as a product of individual oocysts containing genomically heterogeneous sporozoites [4]. This process is driven by the PyMOI library for Python3 and the R library moimix, which carry out haplotype analysis using the full SNP repertoire [17]. Datasets are subjected to variant calling to identify SNPs using FreeBayes (process 2.1) [18]. The full repertoire of whole-genome SNPs (wgSNPs) across each sample is then used to construct a tree and principal co-ordinate analysis (PCoA) clustering plots representing distance in SNP space (process 2.2). SNP space plots are generated using Python libraries (scipy and matplotlib) [19,20]. Finally, reports pooling relevant data for the entire run (process 2.4), along with more granular reports for each sample (process 2.3), are constructed. These reports contain a table of mapping statistics, total and unique SNP count for each sample and data produced from the m.o.i. analysis (process 2.1) and phylogenetic analysis (process 2.2).

All relevant data are deposited in an output directory, the structure for which can be found in File S1, available in the online Supplementary Material.

#### Reporting

Two kinds of reports are produced by Parapipe. First, a report is produced for each sample, containing a table detailing the results of quality control, mapping, m.o.i. analysis and SNP analysis, along with phylogenetic plots for SNP distance. A further report is produced, which pools all relevant data for the entire run. The sample reports are provided in PDF format. The run report is provided in both PDF and HTML formats. JSON files, which are machine readable, for each report are also provided to the user in the output run directory. This run report includes a link to the MultiQC report, which details results from the quality control process.

### Evaluation

#### Read simulation

All reads were simulated using ART v2.5.8 [21] using the Illumina HiSeq 25 (HS25) profile with a read length of 150 bp and an insert size of 200 bp. Reads were simulated as pairs, with the average quality score of all reads set to 30. Reads were simulated using the updated *C. parvum* Iowa II-ATCC reference genome (GenBank assembly GCA_015245375.1) [22].

#### Module testing

Module testing was carried out using simulated raw read data to test each of the functional components of Parapipe, outlined in Fig. 1, to ensure reliability. The most complete chromosome-to-chromosome *C. parvum* reference genome (Iowa II-ATCC) [22] was used as a base sequence into which variants can be introduced and reads simulated to test whether Parapipe can reconstruct known and controlled phylogenetic relationships and heterogeneity profiles. Processes within Parapipe were grouped into testing modules (TMs) according to whether they carry out a single testable function within the pipeline. Each TM within the pipeline was tested using a set of simulated reads designed to test the functional element of that module. Further details of the TMs can be found in Tables S1 and S2. For example, TM05 refers to the module that investigates sample heterogeneity, using the python library PyMOI and the R package moimix (processes 1.8–2.1). The test dataset was simulated using the *C. parvum* reference genome (Iowa II-ATCC) as a backbone to construct a read set covering the genome to 10x in BAM format, and either 100 (representing a low level of heterogeneity) or 1,000 (representing a moderate level of heterogeneity) SNPs were introduced at equally distributed random locations across the genome to meet specified allele frequencies.

Table S2 details the simulated dataset built to test the capacity of Parapipe to compare input samples by the presence of SNPs. This dataset was designed in a hierarchical manner: there are two lineage orders, such that each *n*th-order lineage (e.g. L1.2 would be a second-order lineage) exhibits all SNPs of <*n*th-ordered lineages on the same branch (e.g. L1.2 would exhibit all the same SNPs as L1, with a further set of L1.2-specific SNPs). The module used to carry out phylogenomic inference in Parapipe makes no such assumptions about hierarchy or SNP inheritance, since this could result in fallacious evolutionary relationship inference (i.e. it does not necessarily follow that a population with a set of SNPs is the ancestral population of one which also exhibits them in addition to a novel set of SNPs).

Each simulated dataset was run through only the module it was intended to test, with prior processes that may alter or affect the results removed to ensure validity of results. Only processes producing output required to run the test module were executed.

Results from module testing can be found in File S1.

#### End-to-end testing

Testing of the full pipeline as a single unit was carried out using a *C. parvum* dataset consisting of simulated raw read data of both mixed and clonal samples (*n*=22). The dataset consists of three discrete lineages down to the second order, each consisting of a single dataset representing the first-order lineage and five datasets representing second-order lineages (*n*=18). Compound datasets were also constructed, representing a number of first-order lineage mixtures (*n*=4). Datasets were simulated using a depth of 10x.

#### Testing on real data

Parapipe was run on a dataset consisting of 20 *C*. *parvum* whole-genome samples derived from human and animal infections from four European countries belonging to five *gp60* subtypes from *gp60* family IIa. The whole-genome distance matrix was computed from wgSNP data using Hamming distance (a measure of the number of SNPs that differ between two samples). The *gp60* distance matrix was computed by performing a multiple alignment using clustal [23]. Phylogenetic trees were generated from these distance matrices using the neighbour-joining algorithm in python and displayed using the ETE-toolkit [24]. Correlation analysis between two distance measures was carried out using ordinary least squares regression. Statistical analysis and plotting were carried out using Scikit, numpy and pandas [25,27]. A description of the dataset can be found in File S1. A minimum read number threshold of 100,000 was utilized during quality control. The minimum fraction of the reference genome that must be covered for inclusion in phylogenetic analysis was 80% to a depth of at least 5x. For m.o.i. investigation, only alleles with a minimum frequency of 0.05 and covered to a depth of at least 5x were used.

#### Run time

Parapipe processed the 22 samples representing the end-to-end testing dataset (32,838,480 reads total) in 1 h 9 min 11 s (9.2 CPU hours) and a single dataset (1,216,240 reads total) in 5 min 3 s (13 CPU minutes). All testing was carried out on a laptop running Ubuntu v20.04.6 with 11 cores and 30 GB physical memory and using Nextflow version 24.10.1.

#### Comparison against other pipelines

The majority of the pipelines designed to process and analyse pathogen NGS data are specific to viral and bacterial genomes. Due to the domain-specific challenges and needs faced by protozoan genomics, those pipelines are not suitable for processing *Cryptosporidium* NGS data. Nonetheless, the pre-assembly functionality of these pipelines was compared to that of Parapipe.

#### Computational requirements

Parapipe was designed to run on both standard desktop or laptop systems and high-performance supercomputing clusters. The computational demands of running Parapipe will scale with dataset size, but modest systems (e.g. 4–8 CPUs, 16–32 GB RAM) are sufficient for small- to medium-scale analyses. Thread count can be assigned as a parameter using the native Nextflow command line options.

## Results

### End-to-end testing

Running Parapipe on the end-to-end test dataset recovered both the three major lineages (L1, L2 and L3) and the hybrid lineages simulated within this dataset. Results of wgSNP-space analysis showed three major clusters, along with four satellite points (L1∪2, L1∪3, L2∪3 and L1∪2∪3).

**Fig. 2.**
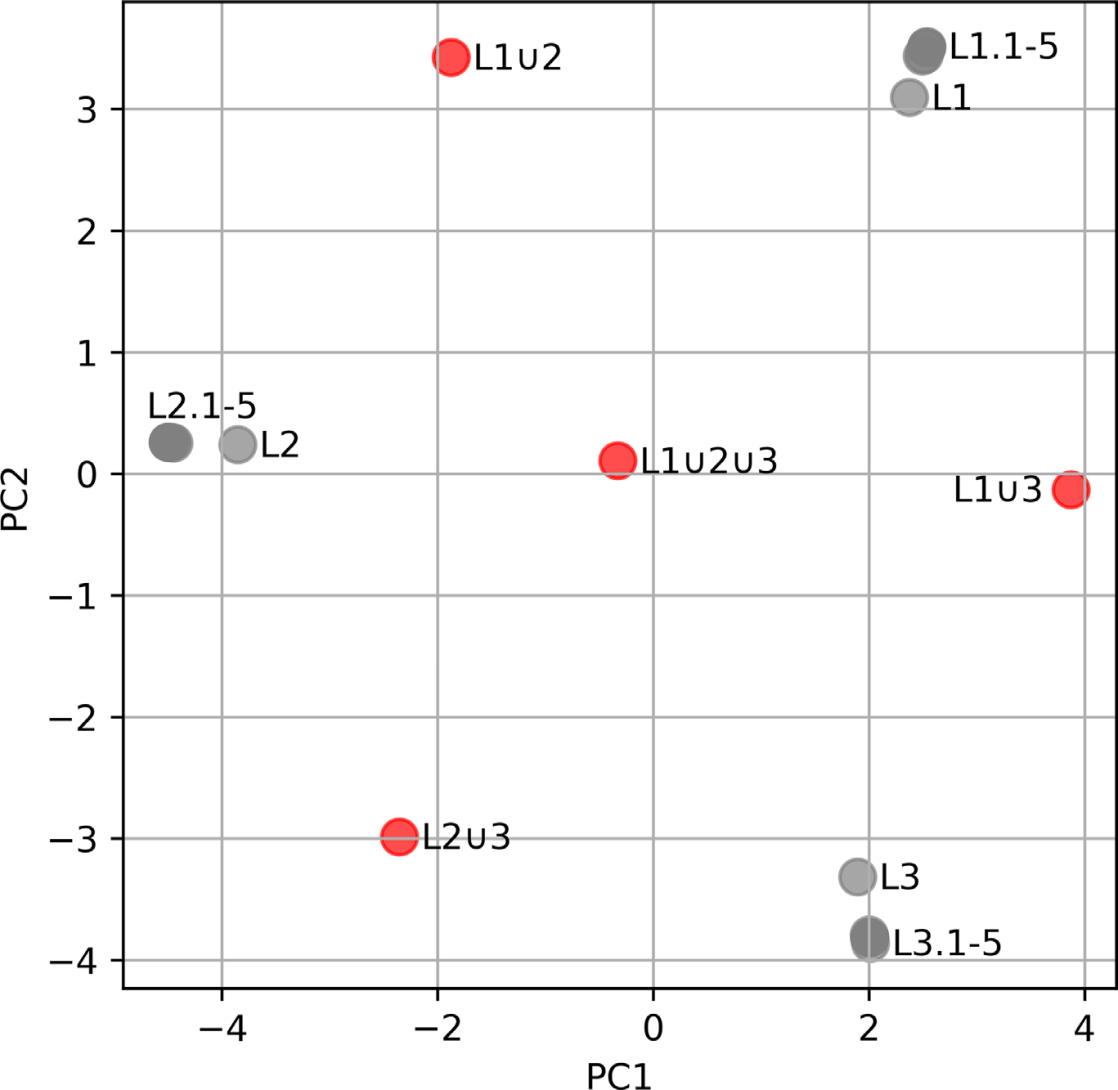
A PCoA plot of the wgSNP distance generated from the end-to-end dataset. PC1 was plotted against PC2, with 74% of the variation space captured across these two components. Marker legend: red=mixed, grey=clonal.

Inspection of the PCoA plot (Fig. 2) revealed more detailed clustering information. Lineages 1, 2 and 3 all form discrete clusters within the dataset. L1∪2 presents as equidistant between lineage clusters 1 and 2, L2∪3 lies between lineage clusters 2 and 3 and L1∪3 lies between lineage clusters 1 and 3. Furthermore, L1∪2∪3 lands directly in the middle of lineage clusters 1, 2 and 3. All satellite samples show a clear indication of sample heterogeneity (*Fws*≥0.95), with L1∪2, L2∪3 and L1∪3 showing a single allele frequency band centring at 0.5, indicating the presence of two distinct populations (each exhibiting an *Fws*≥1.0). Dataset L1∪2∪3 presented with two bands centring at 0.5 and 0.75, indicating the presence of more than two populations (*Fws*≥1.0). All samples covered >99.9% of the reference genome with a median depth of coverage of between 20 and 59x, with high equality of read distribution (Normalized GG area of 0.935–0.96).

### *C. parvum* IIa dataset analysis

**Fig. 3.**
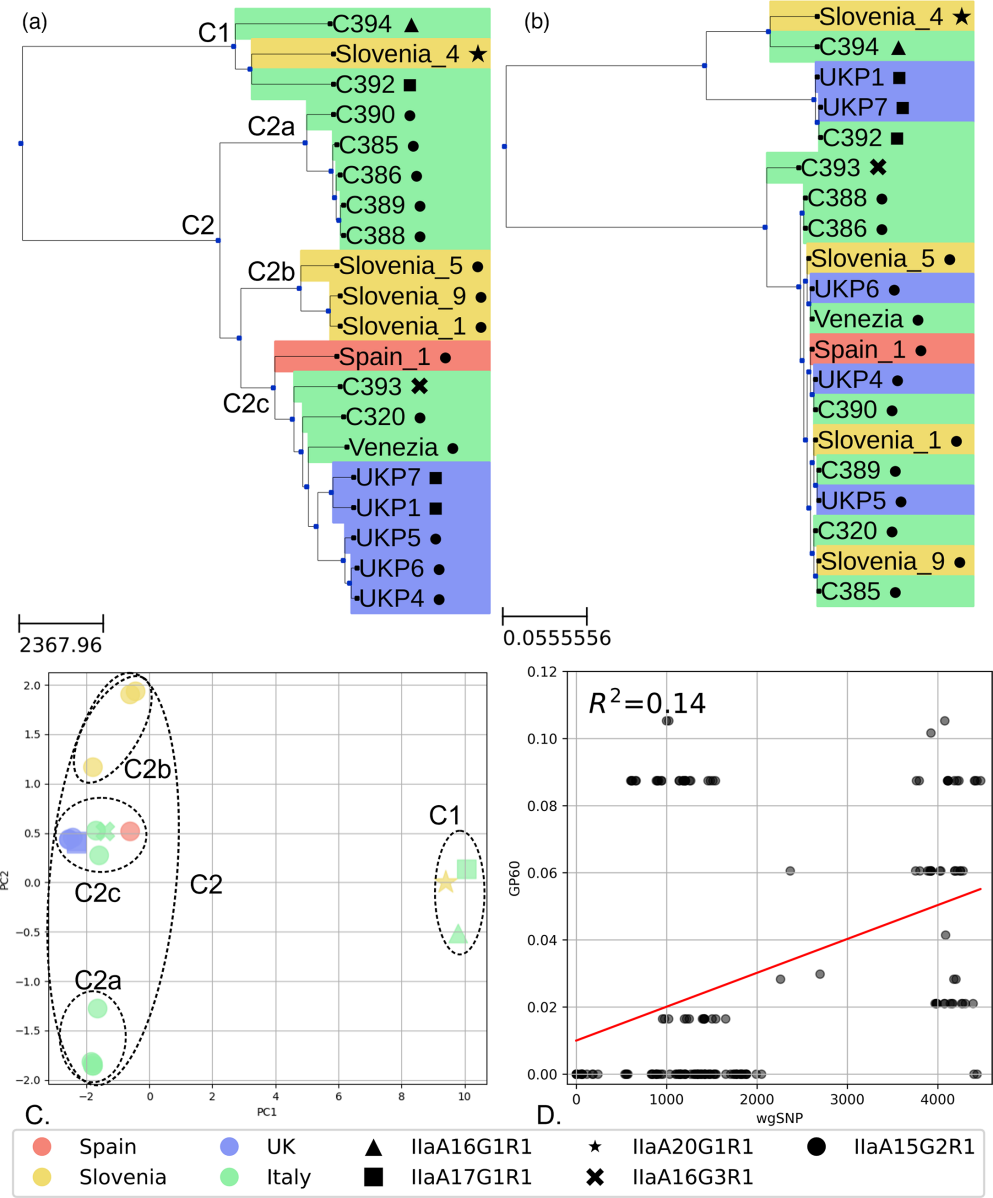
Phylogenetic analysis of this dataset using two typing schemes. (**a**) Phylogenetic tree generated using the wgSNP typing scheme. The scale bar represents Hamming distance. (**b**) Phylogenetic tree generated using the *gp60* sequences. The scale bar represents substitutions per site. (**c**) PCoA carried out using a Hamming distance matrix generated from the wgSNP set. Marker shapes represent *gp60* subtypes. Coloured by country of isolation. (**d**) Correlation analysis of typing schemes generated using the pairwise distance of each pair of samples within the dataset, *R*^2^=0.14, *P*<0.01. Cluster markers are placed by the root node in tree (a) for each cluster and used to annotate the PCoA plot.

Using Parapipe revealed substantial population structure within the *C. parvum* IIa dataset. Analysis by wgSNP Hamming distance indicated the presence of two genetically diverse groups (C1 and C2), which showed association by geographical origin (Fig. 3, panel A). Group C2 could be further subdivided into three subgroups (C2a–c). Diversity within these groups was considerable and showed some correlation within *gp60* sequence typing (Fig. 3, panel B). The *gp60* sequence typing scheme presented two genetically distinct branches. The major branch (most populous) showed extremely limited diversity, indicating they belong to the same *gp60* subtype. The minor (least populous) branch showed substantially more diversity. Analysis using *gp60* sequence typing showed extremely low diversity within the major branch, which comprised primarily of IIaA15G2R1 subtypes, with one IIaA16G3R1 subtype occupying its own minor branch (C393). The minor branch shows high diversity, with the remainder of the non-IIaA15G2R1 samples forming a distal group. Comparison between wgSNP and *gp60* typing showed low correlation (*R*^2^=0.14, *P*<0.01).

## Discussion

### Resolving mixtures in simulated data

In the evaluation phase, the three clonal lineages (L1, L2 and L3), along with their sub-lineages, were resolved by wgSNP Hamming distance analysis. Clustering by SNP distance (Fig. 2) indicated that sub-lineages within this dataset occupied a more distal location in SNP space. Interpretation of clustering results along with the results of m.o.i. analysis (particularly using *Fws*) clearly resolved the clonal components of the simulated mixed samples. Heterogeneity plots can be used to give an indication of the number of populations that exist within a compound dataset, although the interpretation of such plots should be careful and taken in context with other data that may indicate the presence of multiple populations. PCoA plots of SNP distance can be used to reveal whether an ostensibly mixed sample could be a mixture of two known populations; such is the case with L1∪2, which showed strong signals of m.o.i. (*Fws*≥1.0) and can be seen in Fig. 2 to lie between lineages L1 and L2, reflecting the components of this mixture. The same conclusions can be made for L2∪3 (L2 and L3 mixture) and L1∪3 (L1 and L3 mixture). L1∪2∪3 (mixture of L1, L2 and L3) lay equidistant among lineages 1, 2 and 3, demonstrating that Parapipe can be used to resolve the individual components of a triple mixture.

### Parapipe elucidates deep phylogenetic relationships and resolves mixed infections

Results from running Parapipe on the real *C. parvum* IIa genomic dataset (see Table 1) showed substantial within-family diversity, revealed by using wgSNP analysis. The m.o.i. investigation of the dataset provided strong evidence of mixed infection (*Fws*≥0.95) in Venezia (*Fws*=1.0) and weak evidence of mixed infection (*Fws*=0.90–0.95) in C388 (*Fws*=0.922) and C389 (*Fws*=0.924). Corsi *et al*. reported a high m.o.i. (*Fws*≥0.95) in both Venezia and C394 [28]. The apparent clonality of C349 (*Fws*=0.485) here is likely due to the fact that it contains the highest number of unique SNPs (814) in this dataset, making it the most genomically isolated sample. Since *Fws* represents the ratio of within-sample diversity to overall diversity across the dataset, samples from underrepresented populations or those with high genomic isolation tend to exhibit lower *Fws* values. Consequently, their polyclonality may be underestimated. It is therefore essential that interpretation of polyclonality using *Fws* be accompanied by phylogenetic analysis and knowledge of sample origins. The limited metadata associated with these samples did not allow for further investigation into the incidence of m.o.i. across different metadata categories, such as host or country. Phylogenetic analysis showed that there is accordance in the major clade structures between the two typing paradigms (Fig. 3), whereby two clades were defined. However, the membership of these clades differed substantially, potentially leading to inconsistent epidemiological interpretation. Group C1 in particular showed disparate results, whereby C394, C392 and Slovenia_4 all share a group under both typing schemes but were grouped with UKP1 and UKP7 according to *gp60*, whereas wgSNP placed them with other UK isolates. The two typing schemes showed poor correlation within this dataset (*R*^2^=0.14, *P*<0.01), which is revealed by the pairwise correlation plot (Fig. 3, panel D). This result suggests that while *gp60* typing generally reflects the same relative relatedness as whole-genome analysis, it is not a good predictor of the actual genomic distance as measured by wgSNP distance. The *gp60* typing scheme likely only captures a small portion of the overall genomic variation, leading to a weak correlation with wgSNP, and therefore may not provide a comprehensive picture of genomic relatedness. Samples that were closely associated by wgSNP (such as UK isolates) were split into two distinct *gp60* subtypes, IIaA15G2R1 and IIaA17G1R1, indicating a discrepancy between local and global genome mutation and recombination rates. Geographical origins were more robustly recovered by wgSNP than *gp60* typing. This is likely due to the reduced resolution of a single-locus Sanger sequence typing approach, which relies on variation embedded in a very small subset of the *Cryptosporidium* genome. Mutations relevant to elucidating transmission, geographic origin and phenotype, which are embedded within regions not covered by a molecular typing scheme, will not influence the topology of the trees they are used to generate but will be captured by a wgSNP typing approach. Furthermore, a greater degree of discrimination was achieved using wgSNP, resulting in deeper leaf nodes within the tree. Typing by *gp60* places two UK isolates, UKP7 and UKP1, with the Italian isolate C392, since they belong to the same subtype (IIaA17G1R1). However, analysis by wgSNP placed all UK isolates together and showed considerable distance between all UK isolates and C392, indicating substantial genomic divergence between these isolates, which is not captured by *gp60* typing.

**Table 1.** Mapping, m.o.i. and SNP calling results from running the *C. parvum gp60* family IIa dataset through Parapipe

ID	Mean DOC	BOC≥5x	Norm. GG area	*Fws*	SNPs: total (unique)
**C320**	674.6	99.93	0.993	0.744	1,120 (99)
**C385**	619.2	99.95	0.992	0.828	1,376 (4)
**C386**	555.2	99.95	0.992	0.865	1,392 (6)
**C388**	606.7	99.96	0.993	0.922	1,397 (0)
**C389**	640.9	99.95	0.993	0.924	1,403 (3)
**C390**	379.4	99.97	0.991	0.582	1,327 (41)
**C392**	317.8	99.97	0.991	0.491	4,264 (524)
**C393**	450.9	99.97	0.992	0.409	1,182 (90)
**C394**	386.1	99.94	0.991	0.485	4,188 (814)
**Venezia**	973.1	99.89	0.991	1.003	1,147 (100)
**Slovenia_1**	476.2	99.96	0.992	0.815	1,603 (24)
**Slovenia_4**	286.3	99.95	0.991	0.54	4,025 (681)
**Slovenia_5**	351.1	99.96	0.991	0.857	1,155 (34)
**Slovenia_9**	552.8	99.94	0.991	0.813	1,644 (24)
**Spain_1**	564.4	99.92	0.992	0.791	1,314 (449)
**UKP1**	875.7	99.92	0.984	0.732	754 (79)
**UKP4**	260.2	99.37	0.948	0.874	860 (29)
**UKP5**	37.1	98.96	0.945	0.782	857 (65)
**UKP6**	147.4	99.97	0.966	0.881	871 (21)
**UKP7**	104.6	96.96	0.949	0.758	750 (89)

DOC=depth of coverage; BOC≥5x=the percentage of the reference genome covered to a minimum depth of 5x; Norm. GG area=the normalized area under the Gini-Granularity curve [31]; *Fws*=the within-sample F statistic; SNPs: total (unique)=the total and unique number of SNPs detected in this sample.

These results indicated that *gp60* typing can resolve major structures within *Cryptosporidium* population data. However, it was less sensitive than a wgSNP approach at resolving highly granular population structure, particularly across closely related samples. wgSNP analysis indicated more within-clade diversity than the *gp60* typing scheme due to higher resolution. This furnishes the epidemiologist with higher resolution data with which to draw epidemiological inferences and elucidate transmission dynamics, which may be lost with lower resolution typing schemes.

### Parapipe exhibits functional novelty crucial in the analysis of pathogenic protozoans

A comparison between the functionality of Parapipe and similar publicly available pipelines (see Table 2) highlighted that no other pipeline carries out the full repertoire of pre-assembly analysis that Parapipe is capable of. The m.o.i. analysis is an area of analysis missing from other pipelines within the cohort, which were historically developed to analyse subsamples from clonal pathogen populations. Generation of a binary allelic matrix by Parapipe enables a diverse range of downstream analyses, including population structure inference, phylogenetic reconstruction and assessment of genetic diversity. It also supports genome-wide association studies, detection of selective sweeps and admixture and identification of conserved or population-specific genomic regions. Additionally, the matrix serves as a suitable input for machine learning models for genotype–phenotype prediction and classification.

**Table 2.** A comparison between publicly available pipelines for processing pathogen NGS data and carrying out assembly-free analysis

	Parapipe	Bactopia	ASA3P	Nullarbor	ProkEvo	rMAP
Quality control	Y	Y	Y	Y	Y	Y
Read mapping	Y	Y	Y	Y	N	Y
M.o.i. analysis	Y	N	N	N	N	N
SNP detection	Y	Y	Y	Y	N	Y
Assembly-free phylo. analysis	Y	Y	N	Y	N	Y
Multi-sample parallelization	Y	Y	Y	N	Y	Y
Sample report	Y	Y	Y	Y	Y	Y
Multi-sample report	Y	Y	N	Y	Y	Y

While Parapipe is a module and can be extended to support other eukaryotic pathogens, its current implementation is optimized for *Cryptosporidium*. Consequently, fixed parameter selections for processes such as variant calling and m.o.i. analysis have been informed by the genomic characteristics of *Cryptosporidium*, which may not hold for other protists with disparate genomic architecture. Users are advised to carefully consider genome structure and ploidy when applying Parapipe beyond its validated use case.

### Towards the genomic age of cryptosporidiosis surveillance

Currently, due to the expensive and time-consuming nature of carrying out NGS on all clinical samples of *Cryptosporidium*, along with the demonstrable utility of an Multi locus VNTR analysis (MLVA) scheme for identifying and investigating outbreaks [6,8], it is likely that the application of NGS and wgSNP analysis will be limited to selected clinical samples (e.g. those taken from outbreak events) until a scalable and cost-effective laboratory workflow has been designed and implemented. Despite this limitation, it is likely that the future of *Cryptosporidium* typing will utilize NGS technologies, enabled by improvements in sample and library preparation, allowing analyses based on the wgSNPs distance rather than interrogation of a small subset of VNTR loci. This developmental leap has already been made for other pathogens, such as *Salmonella enterica* and *Mycobacterium tuberculosis* [29,30]. Parapipe generates an allelic presence/absence matrix from all samples within the dataset, along with all the data required to perform a thorough quality control assessment for each sample prior to their incorporation into a database of historical samples. Such a database can then be queried to yield key phylogenomic information, informing about the relationship between *Cryptosporidium* samples based on their exhibited allelic repertoire. NGS enables high-resolution and robust analysis of m.o.i., a phenomenon with potentially significant epidemiological implications for *Cryptosporidium*. The m.o.i. analysis can identify mixed infections, potentially signalling exposure to multiple or overlapping transmission sources, differentiate between local transmission and importation and assess its role in the emergence of new genotypes. While the dataset used in this study lacks the epidemiological metadata required to elucidate specific transmission pathways, the m.o.i. findings underscore the complexity of *Cryptosporidium* infections.

Parapipe was written to sit in the middle of a larger workflow that can be employed by public health laboratories, where the first step is *in silico* species identification to facilitate reference genome selection, and downstream analysis includes exposing output to a database and epidemiological hypothesis testing. Parapipe streamlines genomic analysis, providing public health laboratories with a tool to integrate m.o.i. data with geographic, demographic and environmental variables. This integration has the potential to enhance the epidemiological precision of outbreak investigations, facilitating an effective public health response.

## Conclusion

In this article, we have presented Parapipe, a novel bioinformatics pipeline designed to be the foundation of a more complex analytical framework for pathogenic protozoan NGS data. The generation of an allele matrix, along with allele frequency and m.o.i. data, facilitates in-depth phylogenetic analysis. Parapipe streamlines and standardizes a bespoke bioinformatic workflow designed to carry out quality control, heterogeneity and phylogenetic analysis of protozoan NGS data, which are essential parts of any characterization process intended to enable tracking and control of disease in humans or animals. Here, we use both complex simulated datasets and a case study using real data to show that Parapipe is capable of automating quality control and reporting, identifying mixtures within NGS datasets and carrying out both m.o.i. investigation and wgSNP typing for extracting crucial information necessary for determining the phylogenetic relationship between samples. Parapipe represents, to our knowledge, the first publicly available pipeline designed for the analysis and handling of such data and benefits from development within a public health environment, building a modular pipeline that is ISO accreditable by design. Parapipe has been thoroughly tested and validated, using both individual module and end-to-end testing approaches. It is entirely modularized using Nextflow with dependencies managed through the use of Singularity containers. The modularity of the pipeline facilitates the implementation of new functionalities into Parapipe. It is a crucial first step in the development of a full suite of robust, validated bioinformatic tools, which can be used to aid the public health response to *Cryptosporidium*. It represents the first contact point for dealing with clinical (human or animal) or environmental NGS data, giving a detailed but easily digestible overview of the data. Parapipe was designed in accordance with the needs of public health agencies and laboratories.

## Supplementary material

10.1099/acmi.0.000993.v3 Uncited Supplementary Material 1.

## Abbreviations

MLVA: Multi Locus VNTR
NGS: next-generation sequencing
PCoA: principal co-ordinate analysis
TM: testing module
VNTR: variable number tandem repeat
wgSNP: whole-genome SNP
