# Supplementary material for: Parapipe: a pipeline for parasite next-generation sequencing data analysis applied to Cryptosporidium
Source: Access Microbiol. 2025 Aug 8;7(8):000993.v3. doi: 10.1099/acmi.0.000993.v3 (PMC12451305; doi:10.1099/acmi.0.000993.v3)
Supplement: Uncited Supplementary Material 1. [file acmi-7-00993-s001.pdf]

Testing Modules were designated from the processes detailed in Fig. 1. Six discrete modules were defined, which are detailed in Table S1.

Module Code	Processes	Function	Dataset Code	Description	Intended Result	Actual Result
TM01	1.2	Ensure the process which validates the input data functions as intended.	TM01.1	Empty FQ	FAIL	FAIL
			TM01.2	Malformed FQ	FAIL	FAIL
TM02	1.3	Ensure the process which ensures there are enough reads in the input dataset functions as intended.	TM02.1	10% ref 10x cov	FAIL	FAIL
			TM02.2	499,999 read pairs	FAIL	FAIL
			TM02.3	500,000 read pairs	PASS	PASS
			TM02.4	500,001 read pairs	PASS	PASS
TM03	1.4	Ensure the process which filters out short, low quality, and low complexity reads functions as intended.	TM03.1	499,999 read pairs with 2 50n read pairs designed to be filtered out	FAIL	FAIL
			TM03.2	499,999 read pairs with 2 low quality read pairs designed to be filtered out	FAIL	FAIL
			TM03.3	499,999 read pairs with 2 low complexity (poly A) read pairs designed to be filtered out	FAIL	FAIL
TM04	1.7	Ensure mapping statistics are being reported correctly.	TM04.1	1% ref at 25x	Correct mapping statistics	Median DOC: 25.0<br>Ref cov: 0.00998
			TM04.2	10% ref at 25x	Correct mapping statistics	Median DOC: 25.0<br>Ref cov: 0.09998
			TM04.3	25% ref at 25x	Correct mapping statistics	Median DOC: 25.0<br>Ref cov: 0.24998
			TM04.4	50% ref at 25x	Correct mapping statistics	Median DOC: 25.0<br>Ref cov: 0.50063
			TM04.5	75% ref at 25x	Correct mapping statistics	Median DOC: 25.0<br>Ref cov: 0.75063
			TM04.6	100% ref at 25x	Correct mapping statistics	Median DOC: 25.0<br>Ref cov: 0.99998
			TM04.7	100% ref at 1x	Correct mapping statistics	Median DOC: 2..0<br>Ref cov: 0.48645
TM05	1.8-2.1	Ensure heterozygosity is being correctly and robustly reported.	TM05.1	2 populations introduced across 100 sites. AF={0.9, 0.1}	2 clusters centered at correct AF. Fws >= 0.95	2 clusters with correct AF.
			TM05.2	2 populations introduced across 1000 sites. AF={0.9, 0.1}	2 clusters centered at correct AF. Fws >= 0.95	2 clusters with correct AF
			TM05.3	3 populations introduced across 100 sites. AF={0.9, 0.6, 0.1}	3 clusters centered at correct AF. Fws >= 0.95	3 clusters with correct AF
			TM05.4	3 populations introduced across 1000 sites. AF={0.9, 0.6, 0.1}	3 clusters centered at correct AF. Fws >= 0.95	3 clusters with correct AF
TM06	2.2-2.3	Ensure samples are being correctly characterised according to their phylogenetic position.	TM06.1	10 datasets with defined hierarchical lineage structure down to 2nd order	3 clusters each containing 4 samples. Clustered by Lineage, with 1 distal branch to each cluster	Correct tree and cluster topology.

Table S1. Testing modules and datasets used to test Parapipe. AF=allele frequency, as a ratio of reference to alternative allele at a given site. Results of Module testing are included in the Actual Result column.

Order 1 Lineage	Description	Order 2 Lineage	Description
L1	5 SNPs on each chromosome.	L1.1	5 further SNPs on each chromosome.
		L1.2	
		L1.3	
		L1.4	
		L1.5	
L2	5 SNPs on each chromosome.	L2.1	5 further SNPs on each chromosome.
		L2.2	
		L2.3	
		L2.4	
		L2.5	
L3	5 SNPs on each chromosome.	L3.1	5 further SNPs on each chromosome.
		L3.2	
		L3.3	
		L3.4	
		L3.5	

Table S2. Simulated datasets used to test module TM06.

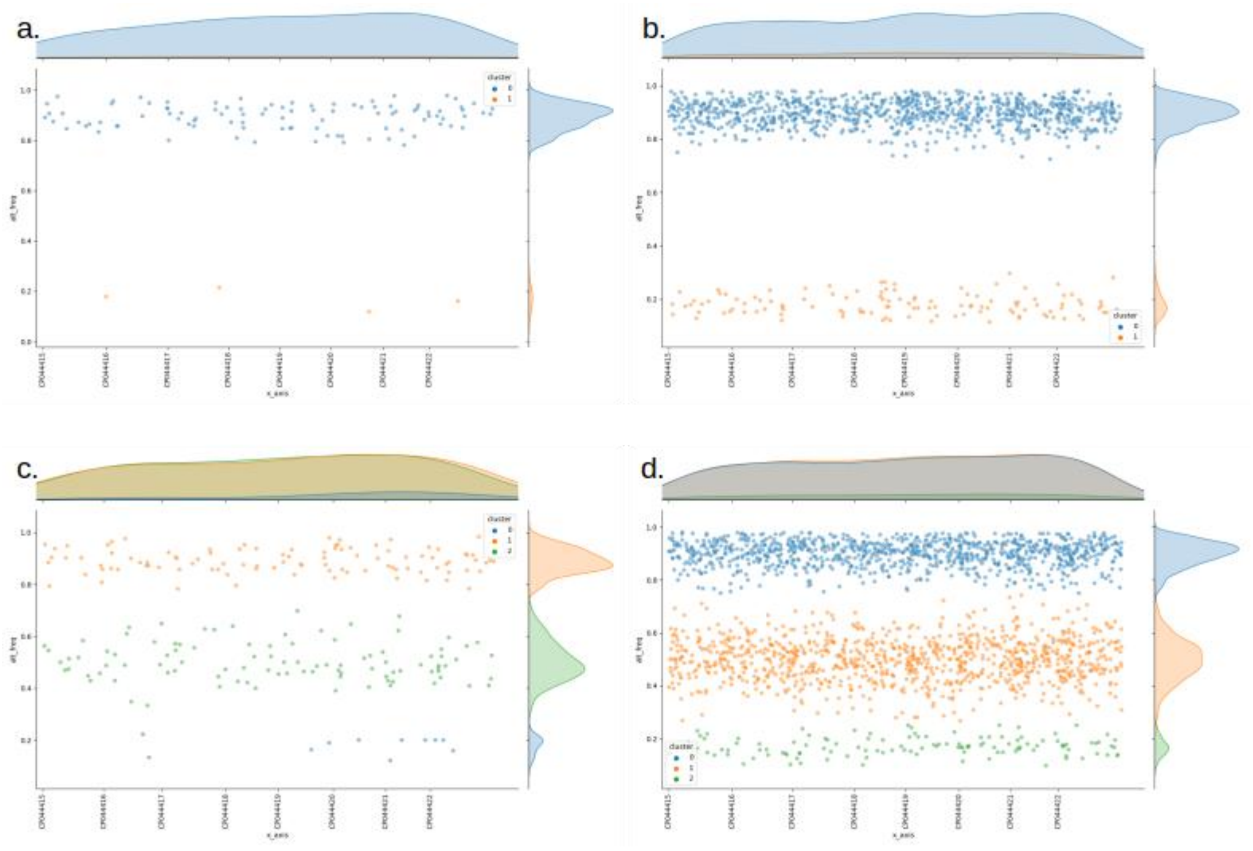

Figure S1. MOI plots generated from running testing module TM05 using the dataset TM05.1 - TM05.4.

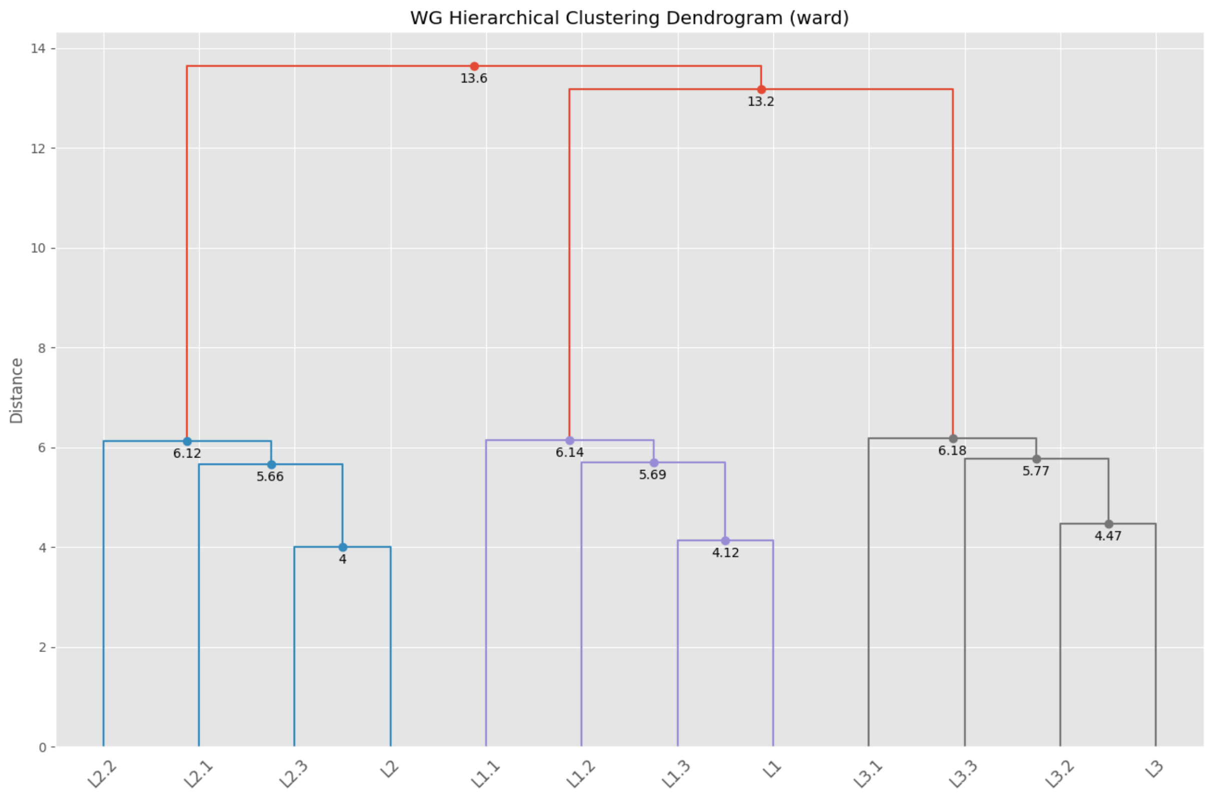

Figure S2. A wgSNP tree generated by running testing module TM06 on test dataset TM06.1.

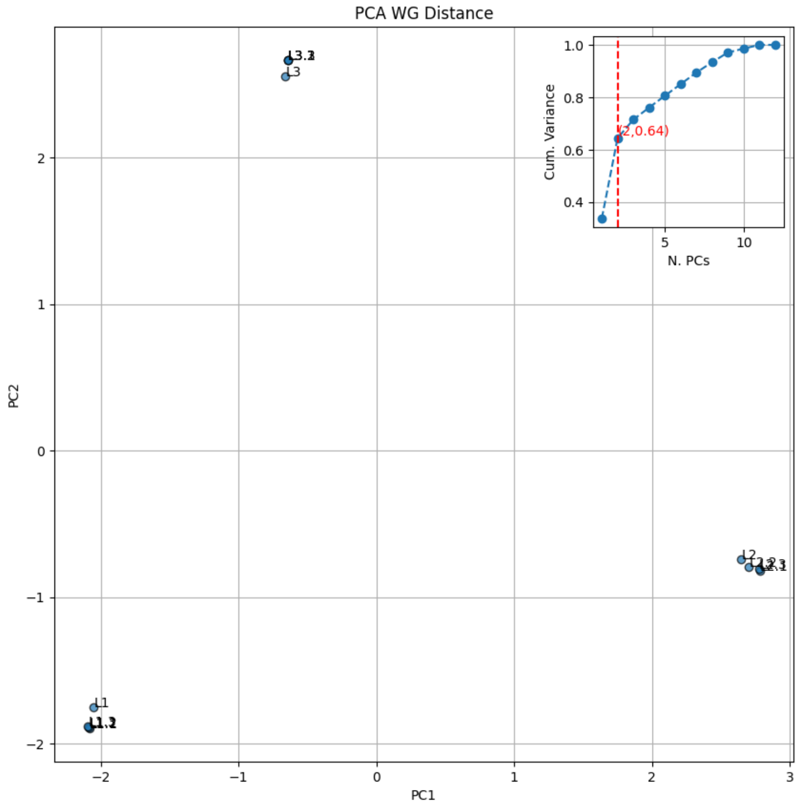

Figure S3. A PCoA plot of wgSNP distance generated by running testing module TM06 on test dataset TM06.1

ID	Mean DOC	Median DOC	BOC>= 5x	GG area	Norm GG area	Av. Qual	Av. Insert Size	Fws	SNPs: total (unique)
**L3.1**	19.8	20	99.93	0.102	0.935	36.4	199.3	0	78 (42)
**L2.4**	19.8	20	99.92	0.103	0.935	36.4	199.3	0.002	81 (40)
**L1.4**	19.8	20	99.92	0.101	0.935	36.4	199.3	0	74 (37)
**L3**	19.8	20	99.93	0.102	0.935	36.4	199.3	0.003	36 (0)
**L1.1**	19.8	20	99.92	0.102	0.935	36.4	199.3	0	76 (38)
**L3.5**	19.8	20	99.91	0.102	0.935	36.4	199.3	0	74 (38)
**L1**	19.8	20	99.93	0.102	0.935	36.4	199.3	0	38 (0)
**L3.3**	19.8	20	99.92	0.102	0.935	36.4	199.3	0.004	72 (36)
**L2.2**	19.8	20	99.92	0.102	0.935	36.4	199.3	0	80 (38)
**L2.5**	19.8	20	99.93	0.102	0.935	36.4	199.3	0.002	80 (38)
**HL1-2**	39.7	39	99.99	0.072	0.953	36.4	199.3	1.119	80 (0)
**L2.1**	19.9	20	99.92	0.102	0.935	36.4	199.3	0	80 (38)
**L2**	19.8	20	99.92	0.102	0.935	36.4	199.3	0	42 (0)
**L2.3**	19.9	20	99.91	0.102	0.935	36.4	199.3	0	78 (36)
**L3.2**	19.8	20	99.93	0.101	0.935	36.4	199.3	0	74 (38)
**HL2-3**	39.7	39	99.99	0.072	0.952	36.4	199.3	1.087	78 (0)
**L1.3**	19.8	20	99.91	0.101	0.935	36.4	199.3	0	75 (37)
**L1.2**	19.8	20	99.92	0.102	0.935	36.4	199.3	0	75 (37)
**HL1-3**	39.7	39	99.99	0.072	0.952	36.4	199.3	1.107	74 (0)
**L1.5**	19.9	20	99.91	0.102	0.935	36.4	199.3	0	69 (31)
**HL1-2-3**	59.5	59	100	0.059	0.96	36.4	199.3	1.008	115 (0)
**L3.4**	19.8	20	99.93	0.101	0.935	36.4	199.3	0	74 (38)

Table S3. The table of mapping statistics for all samples presented in the collated run report.

```
.
└── My_Run_Directory/
    ├── bams/
    │   ├── sample1_grouped.bam
    │   ├── sample2_grouped.bam
    │   ├── sample3_grouped.bam
    │   └── ...
    ├── sample1/
    │   ├── sample1.err
    │   ├── sample1_report.json
    │   ├── sample1_report.pdf
    │   ├── MOI/
    │   │   └── ... heterozygosity data
    │   ├── preprocessing/
    │   │   └── ... mapping and QC data
    │   ├── raw_read_QC_reports/
    │   │   └── sample1_fastp.json
    │   └── VariantAnalysis/
    │       └── SNP/
    │           └── sample1.vcf.gz
    ├── sample2/
    │   └── ...
    ├── sample3/
    │   └── ...
    ├── ...
    ├── mapping_stats/
    │   ├── sample1_mapstats.json
    │   ├── sample2_mapstats.json
    │   ├── sample3_mapstats.json
    │   └── ...
    ├── multiqc_report.html
    ├── Parapipe_report.html
    ├── Parapipe_report.pdf
    ├── phylo/
    │   ├── allele_matrix.csv
    │   ├── sample1.snps.bed
    │   ├── sample2.snps.bed
    │   ├── sample3.snps.bed
    │   └── ...
    └── REFDATA/
        ├── ref.fasta
        └── ref.gff
```

Figure S4. The directory structure for a Parapipe run output directory. The run report is captured in the Parapipe_report.html file, and sample report pdfs deposited in their sample directories.

ID	gp60 Subtype	Country	BioProject	Accession	Study
**C393**	IIaA16G3R1	Italy	PRJNA633764	SRR11817809	Corsi et al. (2023)
**C392**	IIaA17G1R1	Italy	PRJNA633764	SRR11817810	Corsi et al. (2023)
**C390**	IIaA15G2R1	Italy	PRJNA633764	SRR11817812	Corsi et al. (2023)
**C389**	IIaA15G2R1	Italy	PRJNA633764	SRR11817813	Corsi et al. (2023)
**C388**	IIaA15G2R1	Italy	PRJNA633764	SRR11817814	Corsi et al. (2023)
**C386**	IIaA15G2R1	Italy	PRJNA633764	SRR11817815	Corsi et al. (2023)
**C385**	IIaA15G2R1	Italy	PRJNA633764	SRR11817816	Corsi et al. (2023)
**Venezia**	IIaA15G2R1	Italy	PRJNA633764	SRR11817817	Corsi et al. (2023)
**C394**	IIaA16G1R1	Italy	PRJNA633764	SRR11817821	Corsi et al. (2023)
**C320**	IIaA15G2R1	Italy	PRJNA633764	SRR11817823	Corsi et al. (2023)
**Spain_1**	IIaA15G2R1	Spain	PRJNA634014	SRR11818073	Corsi et al. (2023)
**Slovenia_9**	IIaA15G2R1	Slovenia	PRJNA634014	SRR11818074	Corsi et al. (2023)
**Slovenia_5**	IIaA15G2R1	Slovenia	PRJNA634014	SRR11818076	Corsi et al. (2023)
**Slovenia_4**	IIaA20G1R1	Slovenia	PRJNA634014	SRR11818077	Corsi et al. (2023)
**Slovenia_1**	IIaA15G2R1	Slovenia	PRJNA634014	SRR11818079	Corsi et al. (2023)
**UKP4**	IIaA15G2R1	UK	PRJNA253843	SRR6147581	Hadfield et al. (2015)
**UKP5**	IIaA15G2R1	UK	PRJNA253843	SRR6147587	Hadfield et al. (2015)
**UKP6**	IIaA15G2R1	UK	PRJNA253843	SRR6147945	Hadfield et al. (2015)
**UKP7**	IIaA17G1R1	UK	PRJNA253843	SRR6147964	Hadfield et al. (2015)
**UKP1**	IIaA17G1R1	UK	PRJNA253843	SRR6871415	Hadfield et al. (2015)

Table S4. The dataset consisting of *C. parvum* samples belonging to gp60 subtype family IIa.
